# Workplace Harassment and Consideration of Leaving Surgical Practice: A Nationwide Survey by the Japanese Society of Gastroenterological Surgery

**DOI:** 10.1002/ags3.70274

**Published:** 2026-09-07

**Authors:** Keisuke Kurimoto, Tsutomu Fujii, Yoshihiro Shirai, Atsuro Fujinaga, Masayuki Fukumoto, Sonoko Ishida, Koya Hida, Chie Tanaka, Tsunekazu Mizushima, Sachiyo Nomura, Ken Shirabe

**Affiliations:** ^1^ Department of Surgery Nagoya University Hospital Aichi Japan; ^2^ Department of Surgery and Science, Faculty of Medicine, Academic Assembly University of Toyama Toyama Japan; ^3^ Division of Hepatobiliary and Pancreatic Surgery, Department of Surgery The Jikei University School of Medicine Tokyo Japan; ^4^ Department of Gastroenterological and Pediatric Surgery Oita University Faculty of Medicine Oita Japan; ^5^ Department of Surgery, Nagasaki University Graduate School of Biomedical Sciences Nagasaki University Hospital Nagasaki Japan; ^6^ Department of Surgery Kita‐Harima Medical Center Hyogo Japan; ^7^ Department of Surgery Kyoto University Kyoto Japan; ^8^ Department of Colorectal Surgery Dokkyo Medical University Tochigi Japan; ^9^ Clinical Pharmaceutical Sciences Hoshi University Tokyo Japan; ^10^ Division of Hepatobiliary and Pancreatic Surgery, Department of General Surgical Science, Graduate School of Medicine Gunma University Gunma Japan

**Keywords:** gastroenterological surgeons, gender disparity, intention to leave, workforce sustainability, workplace harassment

## Abstract

**Background:**

Workplace harassment in surgical settings is a critical issue affecting surgeon well‐being, workforce sustainability, and patient safety. However, nationwide data focusing on gastroenterological surgeons remain limited.

**Objective:**

To assess prevalence and characteristics of workplace harassment among gastroenterological surgeons in Japan, examine its association with consideration of leaving surgical practice, and evaluate the need for institutional countermeasures by the Japanese Society of Gastroenterological Surgery (JSGS).

**Methods:**

A nationwide, cross‐sectional, anonymous web‐based survey was conducted among JSGS members between November 2024 and January 2025. The questionnaire assessed experiences and observations of five categories of harassment, power, sexual, maternity/paternity, academic, and surgery‐related harassment during the preceding 10 years. Associations with consideration of leaving the profession and opinions on JSGS‐led countermeasures were also evaluated.

**Results:**

A total of 1967 surgeons (response rate, 13.0%) participated. Overall, 66% reported experiencing at least one type of harassment, and 88% reported witnessing harassment in the workplace. Female surgeons reported higher exposure to all harassment types, particularly sexual harassment (55% vs. 8%) and maternity/paternity harassment (24% vs. 4%). Among those who experienced harassment, 56% reported having considered leaving their profession. More than 70% of respondents expressed the need for institutional countermeasures by the JSGS to address all types of harassment.

**Conclusion:**

Workplace harassment is highly prevalent among gastroenterological surgeons in Japan and is associated with consideration of leaving the profession. These findings suggest that harassment represents not only an interpersonal issue but also a structural challenge that may influence the long‐term sustainability of the surgical workforce.

## Graphical abstract

In this nationwide survey of 1967 members of the Japanese Society of Gastroenterological Surgery, 66% reported experiencing workplace harassment, and 56% of those affected had considered leaving surgical practice. These findings highlight the need for sustained, society‐wide efforts to prevent harassment and foster respectful and psychologically safe surgical workplaces.

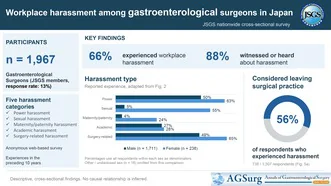

## 1 Introduction

Harassment in the surgical workplace should be understood within the broader context of workplace violence reported among perioperative professionals [1]. It has increasingly been recognized as a critical issue that extends beyond individual interpersonal conflict, with substantial implications for surgeon well‐being, workforce sustainability, and patient safety [2, 3, 4]. Accumulating evidence indicates that exposure to harassment is associated with burnout, deterioration of mental health, and greater intention to leave surgical training or clinical practice [2, 5]. Owing to the traditional hierarchical structure of surgical training, long working hours, high clinical demands and strongly hierarchical organizational cultures, surgeons may be particularly vulnerable to adverse workplace experiences related to harassment [2, 4]. Such environments may also make harassment less likely to become visible and more likely to persist implicitly.

In Japan, surgical specialties face increasing challenges in terms of workforce sustainability. In particular, the number of applicants for surgical training programs has shown a declining trend in recent years, despite an overall increase in the number of interns, thus raising concerns regarding the future availability of surgeons [6, 7, 8, 9]. Within this broader context, gastroenterological surgery is not exempt from this trend. Indeed, a gradual decline in the number of gastroenterological surgeons has also been observed, as reflected by year‐by‐year decreases in membership reported by the Japanese Society of Gastroenterological Surgery (JSGS) [10], despite sustained demand for surgery driven by an aging society [11]. In addition to excessive workload and work–life balance issues, concerns regarding harassment may also influence career choice and career continuity. A 2023 Health Labour Sciences Research report in Japan [12] indicated that, among residents who had originally intended to pursue a surgical career, one of the reasons for ultimately not choosing surgery was the perception that harassment was likely to be common, alongside difficulties in maintaining work–life balance and the image of surgery as involving excessively demanding work. These findings suggest that harassment may constitute a substantial psychological barrier, particularly for young physicians in the early stages of career development.

Furthermore, female physicians have been reported to be affected by gender‐based harassment as well as harassment related to pregnancy, childbirth, and childcare, all of which may influence continued employment and specialty choice [12]. In gastroenterological surgery, where female surgeons remain underrepresented, such issues may also constitute barriers to career development. In addition, a recent nationwide survey conducted by the Japan Surgical Society reported that approximately 40% of young surgeons who had completed surgical training programs had experienced some form of harassment from supervising surgeons, and nearly 30% had considered leaving their training program [7]. Harassment accounted for more than half of the reasons for considering withdrawal, alongside concerns regarding quality of life and work–life balance. Nevertheless, robust nationwide data focusing specifically on gastroenterological surgeons, particularly regarding the prevalence and impact of harassment, remain limited. In response to these circumstances, the JSGS established a dedicated harassment countermeasures committee to address harassment as an organizational and structural issue rather than as isolated incidents. Through discussions within the society and feedback from its members, it became evident that a systematic understanding of the prevalence, types, and consequences of harassment was essential for developing effective countermeasures. Consequently, a society‐led nationwide questionnaire survey targeting gastroenterological surgeons was designed and conducted.

The aim of the present study was to comprehensively assess the experiences and observations of harassment among gastroenterological surgeons in Japan, to examine the association between different types of harassment and consideration of leaving surgical practice, and to describe the views of surgeons on institutional countermeasures by the professional society.

## 2 Methods

### 2.1 Study Design and Participants

This nationwide, cross‐sectional, anonymous, web‐based survey was conducted among JSGS physician members from November 20, 2024, to January 7, 2025. Eligible participants were JSGS members aged 65 years or younger at the time of the survey.

### 2.2 Survey Procedures and Measures

The survey link was distributed to 15 112 JSGS members via the Society's e‐mail newsletter sent by the secretariat and responses were collected anonymously using Google Forms (Google LLC). No restrictions were placed on sex, work style, institution type, or specialist certification. Prenotification and reminder emails were sent during the survey period to encourage participation.

Respondent characteristics (sex, year of medical school graduation, current position, institution type, and workplace region) were assessed using the questionnaire. The questionnaire included items on self‐reported experiences of and witnessing harassment during the preceding 10 years, including type‐specific experiences. Because this was the first nationwide survey conducted by the JSGS on workplace harassment, a 10‐year recall period was selected to comprehensively capture a broad range of harassment experiences and establish a baseline for future repeated surveys. The questionnaire also assessed the perceived impact on consideration of leaving the profession and opinions regarding potential JSGS measures to address harassment. For each harassment category, respondents were also asked about consultation experiences, destinations, timing (career stage), frequency, setting (e.g., institution type, location, and whether it occurred during or outside working hours), characteristics of the perpetrator (e.g., position and approximate age difference relative to the respondent), and the behaviors involved. The survey was conducted using a Japanese questionnaire, and an English translation of the questionnaire is provided in Appendix S1.

For descriptive analyses, respondents who reported experiencing at least one of the five harassment categories were classified as having experienced “Any harassment,” and those who reported witnessing at least one category were classified as having witnessed “Any harassment.”

### 2.3 Harassment Definitions

The survey was used to assess five harassment categories: power harassment, sexual harassment, maternity/paternity harassment, academic harassment, and surgery‐related harassment. Harassment categories were defined with reference to governmental guidance issued by the Ministry of Health, Labour and Welfare and the Ministry of Education, Culture, Sports, Science and Technology in Japan and were adapted to the surgical workplace context.

Additionally, we defined surgery‐related harassment as harassment occurring in surgical care settings (e.g., intraoperative and perioperative management) that exceeds what is necessary and appropriate for work and harms the work environment. This category was introduced to capture inappropriate behaviors occurring in the surgical workplace. It was not intended to be mutually exclusive with other harassment categories, particularly power harassment, and partial overlap between these categories was anticipated. All harassment categories were assessed based on each respondent's subjective perception.

### 2.4 Statistical Analysis

Categorical variables are presented as number with percentages (%), and continuous variables are presented as medians with interquartile ranges (IQRs). Percentages were calculated using the number of respondents who answered each item as the denominator. For figures that describe the prevalence of harassment, “Prefer not to answer” and, where applicable, “Do not know” responses were presented as separate categories.

“Any harassment” was derived from the five categories and coded using a hierarchical rule. Respondents were classified as “yes” if they answered “yes” to at least one category. If not, then they were classified as “no” if they answered “no” to at least one category. Respondents who selected only “do not know” or “prefer not to answer” across all categories were classified as “prefer not to answer.” The same rule was applied to the composite variable “any witnessed harassment.”

The analyses were primarily descriptive, and stratified summaries were prepared by sex and age group. For the analysis of the perceived impact of harassment on career continuation and consideration of leaving the profession, the percentages were calculated among respondents who reported the relevant harassment category and answered the corresponding impact item. Statistical analyses were performed using SPSS version 31.0 (IBM SPSS Statistics, Armonk, NY, USA).

### 2.5 Ethics Considerations

The survey was conducted anonymously, and no information that could identify individual participants was collected. The first page of the questionnaire explained the study purpose and explained that participation was voluntary, with no penalty for nonparticipation. It also stated that only aggregated data would be used for academic purposes. The study protocol was reviewed and approved by the Ethics Committee of the University of Toyama Medical Research Ethical Review Board (reference no.: H2024040). Informed consent to participate was considered to have been obtained through completion and submission of the questionnaire.

## 3 Results

### 3.1 Respondent Characteristics

Table 1 summarizes the characteristics of the 1967 gastroenterological surgeons (13.0%) who completed the survey between November 20, 2024, and January 7, 2025. Of these, 1711 were male (87.0%), 238 were female (12.1%), and 18 preferred not to say (0.9%). The median time since graduation from medical school was 21 years (interquartile range [IQR], 14–29). The largest proportion of respondents had 11–20 years of experience (33.9%), followed by 21–30 years (29.8%) and ≥ 31 years (20.2%). In terms of professional positions, 11.4% were chairpersons, hospital directors, or deputy directors, and 26.6% were department chiefs or deputy chiefs. Professors or associate professors accounted for 6.4% of the respondents. Senior staff surgeons and staff surgeons comprised the largest group (30.3%), followed by lecturers and assistant professors (13.6%). Residents and research fellows represented smaller proportions (4.0% and 3.9%, respectively). Responses were most frequently obtained from private hospitals (21.7%) and national or public university hospitals (21.5%). Although female surgeons and those with 11–30 years since graduation were slightly overrepresented among respondents, the distributions of sex and years since medical school graduation were broadly similar to those of the overall JSGS membership (Table S1).

**TABLE 1:** Background characteristics of respondents.

	Total *n* = 1967 (%)
Sex, (%)	
Male	1711 (87.0)
Female	238 (12.1)
Prefer not to say	18 (0.9)
Years since medical school graduation, years, median (IQR)	21.0 [14.0, 29.0]
Stratified by years since graduation	
≤ 5 years	51 (2.6)
6–10 years	265 (13.5)
11–20 years	666 (33.9)
21–30 years	587 (29.8)
≥ 31 years	398 (20.2)
Professional position	
Chairperson/Hospital director/Deputy director	225 (11.4)
Department chief/Deputy chief	523 (26.6)
Senior staff surgeon/Staff surgeon	596 (30.3)
Resident (including surgical specialty trainee)	79 (4.0)
Professor/Associate professor	126 (6.4)
Lecturer/Assistant professor	267 (13.6)
Research fellow/Graduate student	77 (3.9)
Other	24 (1.2)
Prefer not to say	50 (2.5)
Type of institution	
University hospital (national or public)	423 (21.5)
University hospital (private)	242 (12.3)
National Hospital Organization (NHO)	105 (5.3)
Prefectural hospital	133 (6.8)
Municipal hospital	269 (13.7)
Public hospital (e.g., Japanese Red Cross, Saiseikai)	273 (13.9)
Private hospital	426 (21.7)
Clinic	44 (2.2)
Other	14 (0.7)
Prefer not to say	38 (1.9)
Region of practice	
Hokkaido, Tohoku	200 (10.2)
Kanto	570 (29.0)
Chubu	399 (20.3)
Kinki	387 (19.7)
Chugoku	128 (6.5)
Shikoku	49 (2.5)
Kyushu, Okinawa	196 (10.0)
Overseas	8 (0.4)
Prefer not to say	30 (1.5)

*Note:* This table shows characteristics of survey respondents.

Abbrviation: IQR, interquartile range.

### 3.2 Prevalence of Harassment Among Gastroenterological Surgeons

**FIGURE 1:**
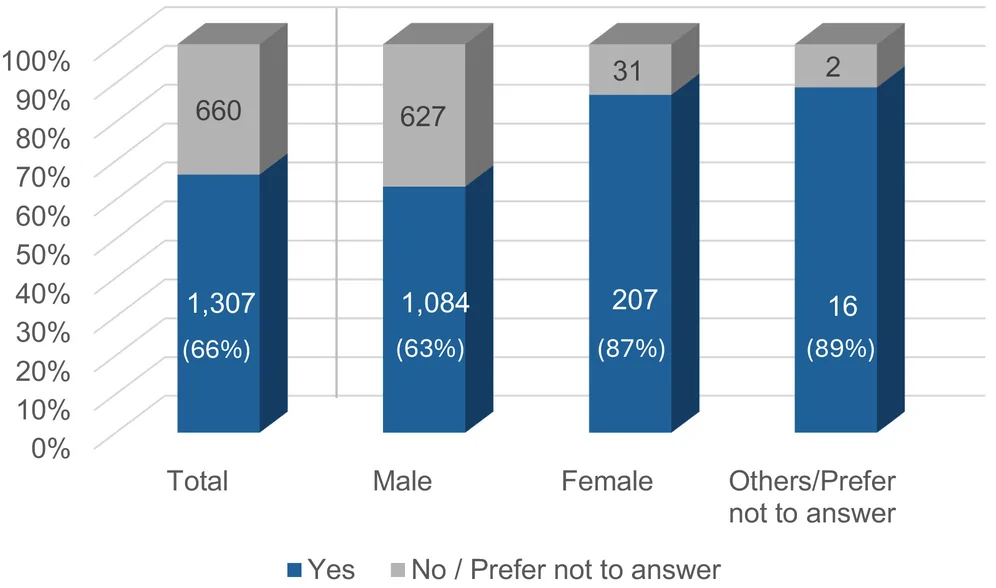
Proportion of gastroenterological surgeons who have experienced any harassment during the preceding 10 years. Overall, 66% of gastroenterological surgeons reported experiencing at least one type of harassment. When stratified by sex, harassment was reported by 87% of female surgeons, compared with 63% of male surgeons.

Overall, 1307 respondents (66%) reported experiencing at least one type of harassment (Figure 1). When stratified by sex, 87% of female surgeons reported experiencing harassment, compared with 63% of male surgeons. These findings indicate that a substantial proportion of gastroenterological surgeons have encountered harassment in their professional environment, although the type and severity of such experiences varied.

### 3.3 Types of Harassment

**FIGURE 2:**
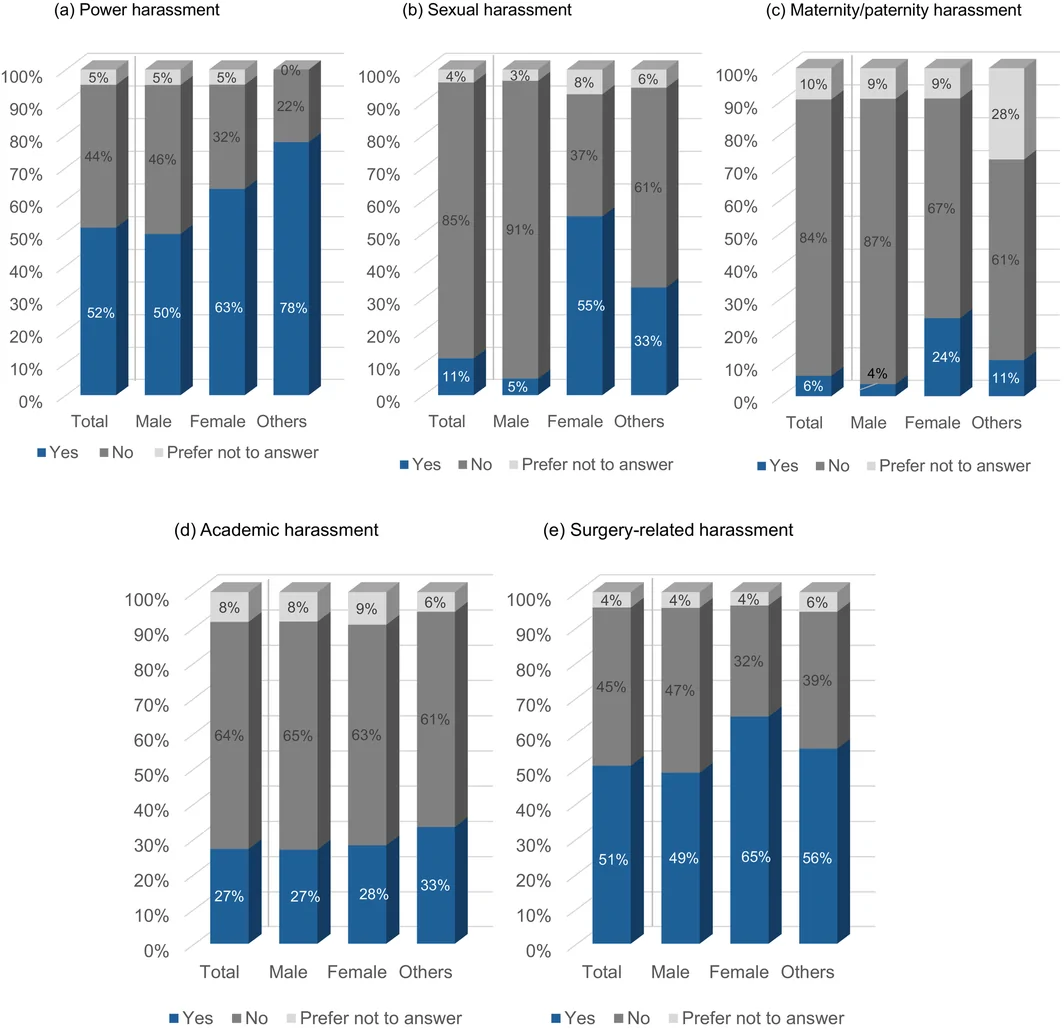
Proportion of gastroenterological surgeons who have experienced various types of harassment during the preceding 10 years, including (a) power harassment, (b) sexual harassment, (c) maternity/paternity harassment, (d) academic harassment, and (e) surgery‐related harassment.

As shown in Figure 2, power harassment (52%) and surgery‐related harassment (51%) were the most frequently reported, each affecting more than half of the respondents. Academic harassment was reported by 27%, whereas sexual harassment and maternity/paternity harassment were reported by 11% and 6%, respectively. In addition to individuals who experienced only a single type of harassment, a substantial proportion of respondents reported exposure to multiple types of harassment, either concurrently or at different time points. For all types of harassment, the proportion of female surgeons reporting exposure exceeded that of male surgeons. This disparity was particularly marked for sexual harassment (55% in females vs. 8% in males) and maternity/paternity harassment (24% vs. 4%). The relatively low overall prevalence of sexual harassment and maternity/paternity harassment reflects the predominance of male respondents. Even for surgery‐related harassment, the prevalence among female surgeons was 16 percentage points higher than that among male surgeons. In contrast, no substantial sex‐based difference was observed for academic harassment.

### 3.4 Proportion of Gastroenterological Surgeons Who Have Witnessed Any Harassment

**FIGURE 3:**
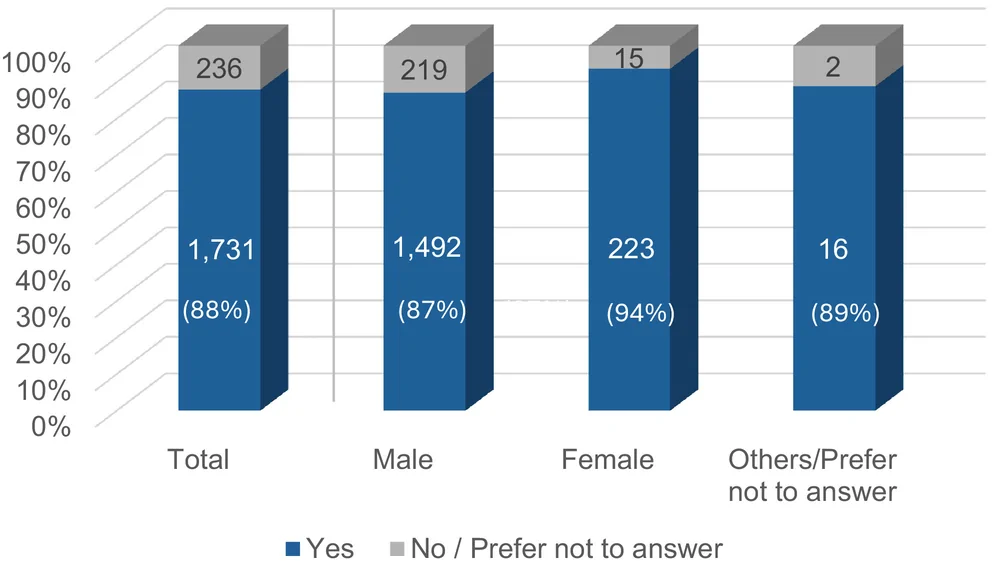
Proportion of gastroenterological surgeons who have witnessed any harassment during the preceding 10 years. Overall, 88% of gastroenterological surgeons reported witnessing at least one type of harassment in the workplace.

Over the past 10 years, 88% of respondents reported having witnessed or heard about at least one type of harassment (Figure 3). This included 87% of male surgeons and 94% of female surgeons. These findings suggest that harassment is not limited to isolated individuals but is widely recognized across the gastroenterological surgical community.

### 3.5 Proportion of Gastroenterological Surgeons Who Have Witnessed Various Types of Harassment

**FIGURE 4:**
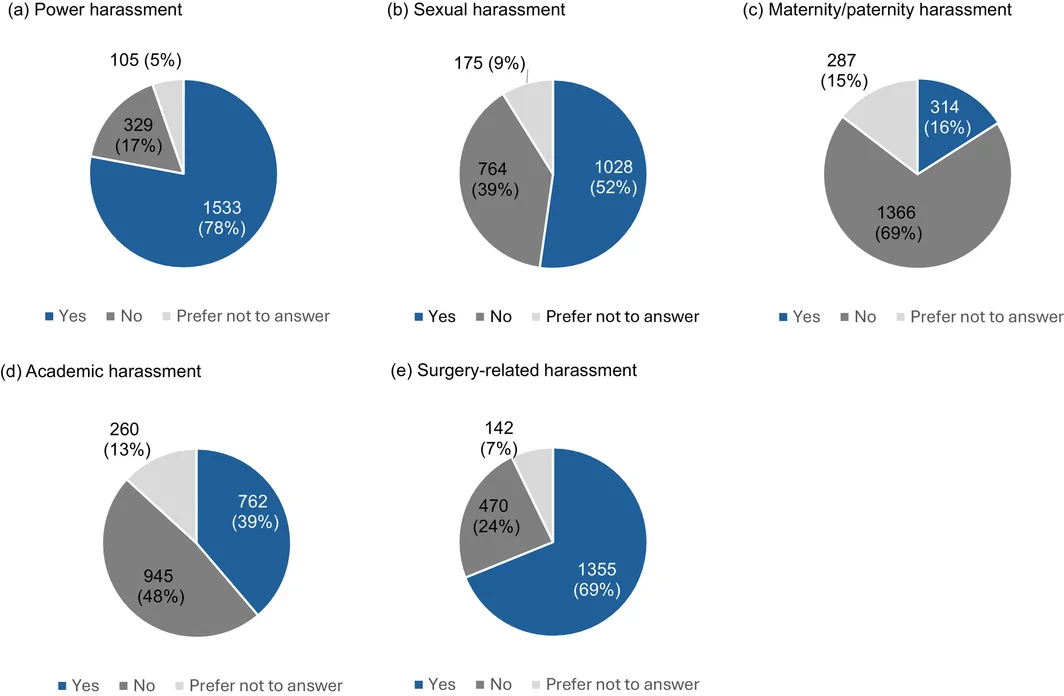
Proportion of gastroenterological surgeons who have witnessed various types of harassment during the preceding 10 years, including (a) power harassment, (b) sexual harassment, (c) maternity/paternity harassment, (d) academic harassment, and (e) surgery‐related harassment.

The most frequently witnessed type was power harassment (78%), followed by surgery‐related harassment (69%), sexual harassment (52%), academic harassment (39%), and maternity/paternity harassment (16%) (Figure 4). For every type of harassment, the proportion of surgeons who witnessed harassment was greater than the proportion who personally experienced it. Notably, more than half of respondents (52%) reported witnessing sexual harassment, indicating that such incidents are not uncommon. In contrast, maternity/paternity harassment was witnessed by 16% of respondents, a lower proportion compared with other types. This may not necessarily indicate lower incidence, but rather that such harassment affects a more limited group and is less visible to others.

### 3.6 Considering Leaving the Profession due to Harassment

**FIGURE 5:**
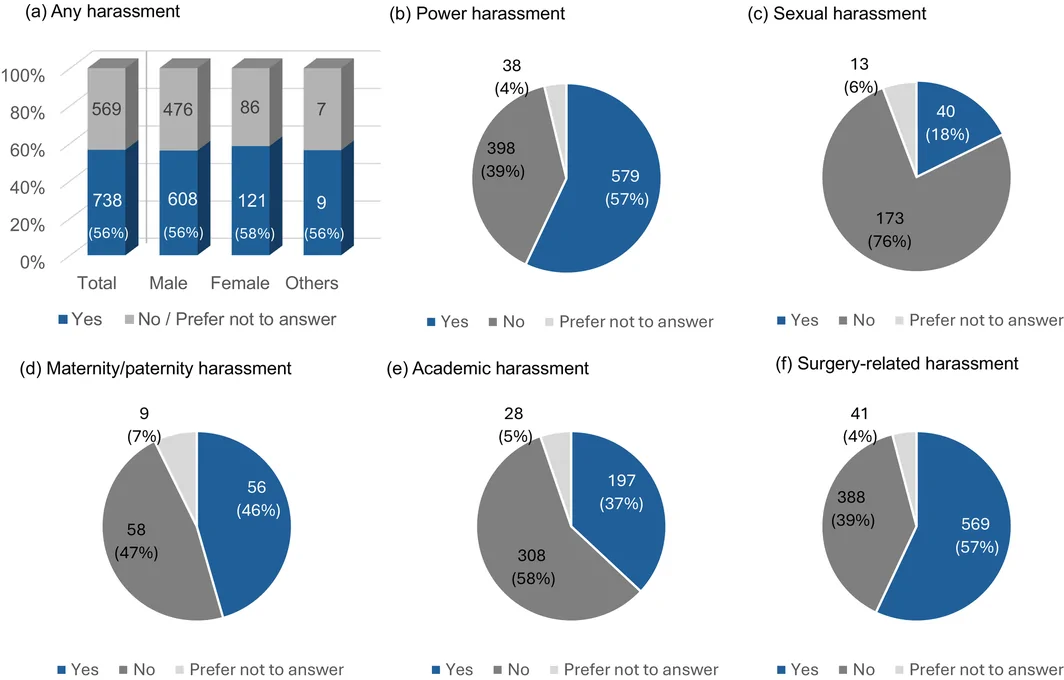
Proportion of gastroenterological surgeons who considered leaving their profession because of harassment, including (a) any harassment, (b) power harassment, (c) sexual harassment, (d) maternity/paternity harassment, (e) academic harassment, and (f) surgery‐related harassment.

Among the gastroenterological surgeons who experienced harassment, 56% (male, 56%; female, 58%) reported that they had considered leaving their profession as a result (Figure 5). With respect to power harassment and surgery‐related harassment, 57% of affected surgeons reported considering leaving their specialty. Similarly, 46% considered leaving because of maternity/paternity harassment. The corresponding proportions were 37% for academic harassment and 18% for sexual harassment.

**FIGURE 6:**
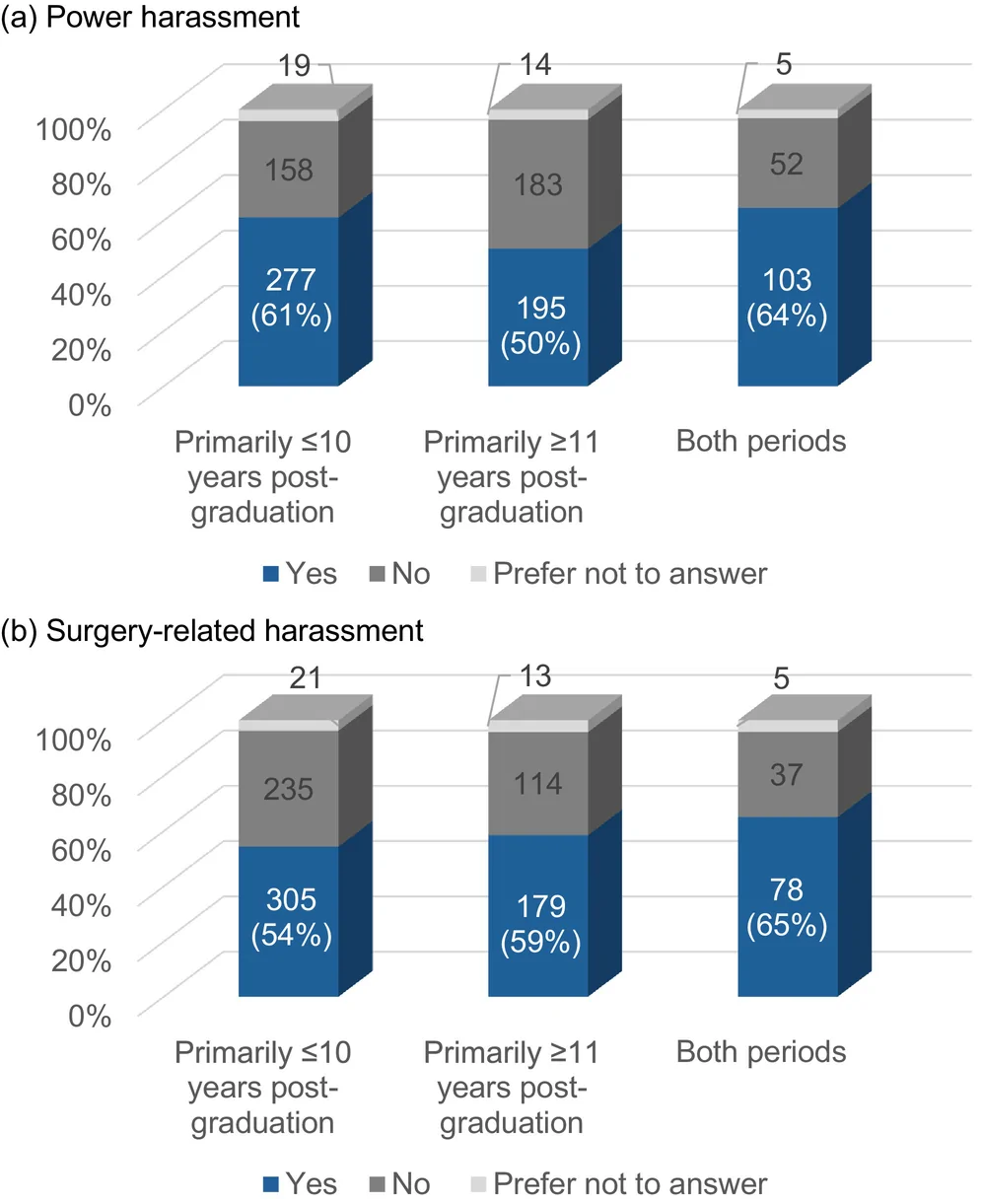
Association between timing of harassment experiences and the proportion of surgeons considering leaving surgical practice because of harassment during the preceding 10 years. (a) Power harassment and (b) surgery‐related harassment were analyzed according to the primary timing of harassment experiences: Primarily within 10 years after graduation, primarily ≥ 11 years after graduation, or across multiple periods.

We further examined the association between the timing of harassment (in relation to years since graduation) and consideration of leaving a surgical career (Figure 6). Among those who experienced power harassment within 10 years of graduation, 61% considered leaving, compared with 50% among those who experienced it more than 10 years after graduation. No significant difference was observed in surgery‐related harassment between early‐career (≤ 10 years) and later‐career (≥ 11 years) groups. Notably, respondents who experienced harassment across multiple time periods were most likely to consider leaving their surgical careers, regardless of the type of harassment.

### 3.7 Requests for Harassment Countermeasures From the JSGS

**FIGURE 7:**
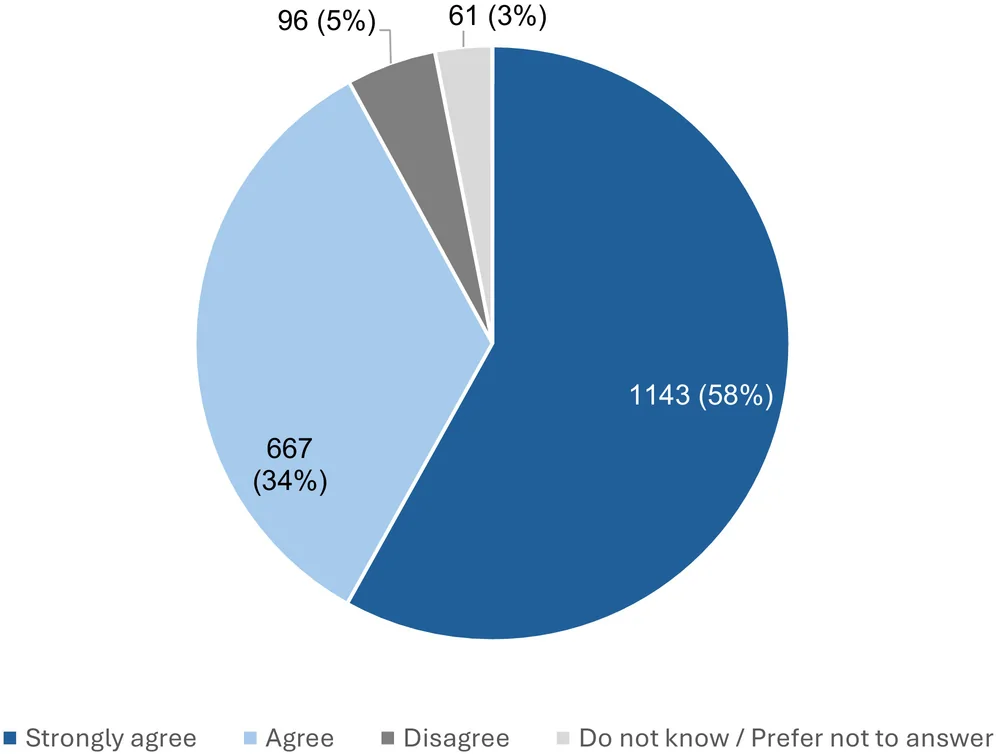
Opinions regarding the need for harassment countermeasures by the Japanese Society of Gastroenterological Surgery (JSGS).

Figure 7 presents the proportion of respondents who indicated that countermeasures against harassment by the JSGS were necessary. A total of 1143 respondents (58%) “strongly agreed,” and 667 (34%) “agreed,” indicating that 92% of respondents supported the need for action by the society. In contrast, only 96 respondents (5%) expressed disagreement. Overall, the results demonstrate a strong demand for proactive involvement by the JSGS and the implementation of concrete measures to address harassment.

## 4 Discussion

In this nationwide survey of 1967 gastroenterological surgeons in Japan, two‐thirds of respondents reported having experienced some form of harassment, and nearly 90% reported witnessing harassment in the workplace. Among those who experienced harassment, more than half considered leaving their profession. Furthermore, 92% of the respondents expressed a clear need for institutional countermeasures by the JSGS, their professional society. These findings indicate that harassment among gastroenterological surgeons is not an isolated interpersonal issue but may represent a broader organizational problem with potential implications for workforce sustainability. In a recent nationwide survey of general workers commissioned by the Japanese Ministry of Health, Labour and Welfare [13], 19.3% and 6.3% of respondents reported experiencing power harassment and sexual harassment, respectively. In contrast, the corresponding proportions in the present survey were 51% and 11%, suggesting that harassment is considerably more prevalent among gastroenterological surgeons than in the general workforce.

The overall prevalence of harassment in our study is consistent with, or higher than, that reported in international studies of surgical trainees and physicians [1, 2, 3, 4, 5]. However, unlike many previous studies focusing primarily on residency training, our survey included surgeons across a wide range of career stages, including senior staff and departmental leaders. The high prevalence observed across career levels suggests that harassment in surgical workplaces may persist throughout professional life rather than being confined to early training years. These findings suggest that harassment may be associated with consideration of leaving surgical practice and may represent a psychological barrier influencing career choice and long‐term career commitment, particularly among younger physicians.

A particularly notable finding was the marked sex disparity in harassment experiences. Female surgeons reported substantially higher exposure to sexual harassment and maternity/paternity harassment and reported higher rates of power and surgery‐related harassment. Although female surgeons constituted a minority of respondents, the disproportionate burden they reported underscores ongoing gender‐related vulnerabilities within surgical culture. These findings align with prior reports demonstrating that female surgeons are at increased risk of gender discrimination and harassment [5, 14], and these findings highlight the importance of structural and cultural reforms to promote equity and inclusivity. In gastroenterological surgery, where female surgeons remain underrepresented, failure to improve such environments may hinder the development of a more diverse and sustainable surgical workforce [15].

Importantly, more than half of surgeons who experienced harassment reported considering leaving their profession. This proportion was particularly high for power harassment and surgery‐related harassment. Given the ongoing decline in the number of applicants to surgical training programs in Japan [6, 7], the association between harassment and consideration of leaving surgery raises concern in an already strained workforce [16]. Previous surveys among Japanese gastroenterological surgeons have also highlighted excessive workload and work–life balance issues as major concerns within the specialty [17]. Notably, respondents exposed to harassment across multiple career stages were most likely to consider leaving surgery, suggesting that repeated or cumulative exposure may be associated with stronger consideration of leaving surgical practice. Harassment should therefore be considered not only an ethical and occupational issue but also an issue with potential implications for workforce policy. Among younger surgeons in particular, inappropriate behaviors occurring within hierarchical educational and operative settings may be perceived as harassment, even when presented in the context of surgical instruction or supervision. Although surgical education sometimes requires strict guidance and tension‐filled situations, educational quality and a healthy work environment should not be considered mutually exclusive.

The concept of surgery‐related harassment requires careful interpretation. During the development of the questionnaire, the authors recognized that this category would inevitably overlap with conventional power harassment. Nevertheless, we considered it important to better understand inappropriate behaviors occurring within the unique environment of the surgical workplace in order to inform future discussion and the development of effective preventive measures. Accordingly, surgery‐related harassment was introduced as a complementary category to capture such behaviors. This category was not intended to be mutually exclusive from power harassment or other forms of harassment, but rather to provide a practical framework for understanding behaviors that may be characteristic of the surgical workplace and informing future preventive measures.

Another striking observation was that witnessing harassment was more common than direct personal experience for all types of harassment. This suggests that harassment may be embedded in workplace culture and normalized within surgical environments [7, 16]. Taken together, these findings suggest that harassment should be considered not only an interpersonal problem but also an organizational issue within the surgical workplace. Organizational approaches, including fostering a respectful workplace culture and psychological safety, may therefore be important components of future prevention strategies. At the institutional level, educational approaches to harassment, including video‐based and simulation‐based training for residents, have also begun to be explored [18, 19]. Preventive efforts should therefore address not only individual incidents but also broader cultural norms.

Our findings also demonstrate a strong demand for institutional action, with 92% of respondents expressing the need for countermeasures by the JSGS. Professional societies may play a critical role in establishing standards, providing education and fostering a culture of zero tolerance. In recent years, the JSGS has actively addressed issues related to work‐style reform, workforce sustainability, and workplace environment through nationwide surveys and policy discussions [20]. Society‐led initiatives are particularly important in hierarchical specialties such as surgery, where institutional responses may be constrained by hierarchical power structures and conflicts of interest. In addition to continued awareness campaigns, future initiatives by the JSGS may include structured anti‐harassment education for trainers and department leaders, periodic nationwide surveys, and systems to recognize and promote institutions that maintain healthy educational and working environments. In Japan, the Japanese Medical Specialty Board has established a consultation pathway for trainees regarding suspected harassment [21], and the Japanese Surgical Society Education Committee U‐40 working group has reported survey‐based analyses of harassment in surgical training [7, 16]. Internationally, formal professional‐society resources addressing harassment‐related behaviors have also been developed [22, 23], and the Anesthesia Patient Safety Foundation has released video‐triggered workshop modules for healthcare workplace violence prevention [24]. Together, these reports support the feasibility of society‐led and simulation‐informed approaches, although evidence for their real‐world effectiveness in surgery remains limited.

This study has several limitations. First, because participation was voluntary, substantial self‐selection bias cannot be excluded. Surgeons with strong opinions or experiences regarding harassment may have been more likely to respond. Comparison with the overall JSGS membership showed broadly similar distributions of sex and career stage (Table S1). However, this does not eliminate the possibility of self‐selection bias inherent in a voluntary survey. Second, harassment was assessed based on subjective perception, which may not necessarily align with legal evaluations or objective factual determinations. In addition, direct observation of harassment and hearing about harassment were assessed within a single item and could not be distinguished in the present analysis. Third, causal relationships between harassment and career decisions cannot be established because of the cross‐sectional design. Furthermore, surgeons who had already left surgical practice or withdrawn from the JSGS were not included in this survey, including those who may have done so because of harassment. Fourth, because the survey was conducted in the context of a professional society actively addressing harassment issues, a framing effect may have influenced respondents' perceptions and responses. Fifth, because respondents were asked to recall experiences over the preceding 10 years, recall bias is unavoidable. During this period, workplace culture, physician work‐style reform, and societal awareness of harassment changed substantially. Consequently, respondents may have evaluated past events according to current norms, and their present assessments may not fully reflect how those events were perceived at the time they occurred. Additionally, as detailed interviews regarding individual cases and objective third‐party evaluations were not conducted, the possibility of some degree of overreporting cannot be ruled out. Because this survey included only JSGS members, the findings may not be directly generalizable to other surgical specialties, nonsurgical disciplines, or healthcare systems outside Japan. Given these points, caution is necessary when interpreting the findings of this study. Nevertheless, the large nationwide sample and the inclusion of surgeons across different career stages provide a broad overview of perceived workplace harassment among gastroenterological surgeons in Japan.

These findings support continued efforts by the JSGS to raise awareness, promote education, and periodically monitor harassment to foster a work environment in which surgeons can practice safely and with confidence. As part of its ongoing efforts, the JSGS issued a formal “Declaration for the Eradication of Harassment” on July 17, 2025, underscoring its commitment to addressing this issue at the society level. The findings of the present survey support both the necessity and validity of this declaration and emphasize the importance of translating the declaration into concrete actions rather than leaving it solely as a statement of principle.

## 5 Conclusion

Harassment was frequently reported among gastroenterological surgeons in Japan and was associated with self‐reported consideration of leaving the profession. The high rate of witnessed harassment further suggests that problematic behaviors may be embedded in workplace culture rather than representing isolated incidents. These findings suggest that harassment should be recognized not merely as an individual interpersonal issue, but also as an organizational challenge that may have implications for the future sustainability of gastroenterological surgery. The JSGS remains committed to continuing society‐led efforts to create a safer and more supportive environment for all surgeons, including younger and female surgeons.

## Author Contributions

**Sonoko Ishida:** methodology, conceptualization, investigation, writing – review and editing. **Tsunekazu Mizushima:** conceptualization, investigation, methodology, writing – review and editing. **Masayuki Fukumoto:** methodology, investigation, conceptualization, writing – original draft, writing – review and editing. **Keisuke Kurimoto:** methodology, investigation, conceptualization, software, data curation, writing – review and editing, writing – original draft, project administration. **Tsutomu Fujii:** methodology, conceptualization, investigation, software, supervision, writing – review and editing, writing – original draft, project administration. **Atsuro Fujinaga:** methodology, conceptualization, investigation, writing – original draft, writing – review and editing. **Yoshihiro Shirai:** methodology, conceptualization, investigation, writing – original draft, writing – review and editing. **Ken Shirabe:** methodology, conceptualization, investigation, writing – review and editing. **Koya Hida:** methodology, conceptualization, investigation, writing – review and editing. **Chie Tanaka:** methodology, conceptualization, investigation, writing – review and editing. **Sachiyo Nomura:** methodology, conceptualization, investigation, writing – review and editing.

## Funding

The authors have nothing to report.

## Disclosure

Tsutomu Fujii, Chie Tanaka, Tsunekazu Mizushima, Sachiyo Nomura, and Ken Shirabe are the Editorial Board members of Annal of Gastroenterological Surgery and the coauthors of this article. To minimize bias, they were excluded from all editorial decision‐making related to the acceptance of this article for publication.

## Ethics Statement

This study was approved by the Ethics Committee of the University of Toyama Medical Research Ethical Review Board (approval no. H2024040) (as noted in the manuscript).

## Consent

Informed consent was considered to have been obtained through voluntary completion and submission of the anonymous questionnaire (as noted in the manuscript).

## Conflicts of Interest

The authors declare no conflicts of interest.

## Supporting information


**Appendix S1:** Survey on harassment in general and gastroenterological surgery.

**Table S1:** Characteristics of respondents and overall JSGS membership.
