# Supplementary material for: Workplace Harassment and Consideration of Leaving Surgical Practice: A Nationwide Survey by the Japanese Society of Gastroenterological Surgery
Source: Ann Gastroenterol Surg. 2026 Sep 7:10.1002/ags3.70274. Online ahead of print. doi: 10.1002/ags3.70274 (PMC13550732; doi:10.1002/ags3.70274)
Supplement: Supplementary file 1 [file AGS3-9999-0-s001.docx]

Appendix S1. Survey on Harassment in General and Gastroenterological Surgery

Survey on Harassment in General and Gastroenterological Surgery

*The Japanese Society of Gastroenterological Surgery is conducting a survey on workplace harassment. The purpose of this survey is not to assign blame for past events but to understand trends in surgical workplaces and use the findings to improve future surgical education. We would greatly appreciate the participation of as many respondents as possible. No individuals or institutions will be identifiable from the responses, so please describe only the incidents themselves. All personal information will be handled strictly and confidentially in the analysis and reporting of the results.*

*Please answer the following questions about your own experiences during the past 10 years in the following five categories: power harassment, sexual harassment, harassment related to pregnancy, childbirth, and childcare (maternity or paternity harassment), academic harassment, and surgery-related harassment. Some questions include specific examples, and answering them may bring back unpleasant memories. Please respond only to the extent that you feel comfortable. The survey will take approximately 20-25 minutes to complete.*

Definitions of Harassment

- **Power harassment**: words or actions arising from a superior relationship* that exceed what is necessary and appropriate for work and thereby harm the person's work environment.
- **Sexual harassment**: a situation in which a person's response to sexual remarks or conduct results in disadvantageous working conditions, or in which such remarks or conduct harm that person's work environment.
- **Harassment related to pregnancy, childbirth, and childcare (maternity or paternity harassment)**: a situation in which words or actions related to pregnancy, childbirth, or childcare harm the work environment of female employees or of male employees whose partners are pregnant, have given birth, or are raising children.
- **Academic harassment**: in educational or research settings, inappropriate remarks, conduct, or supervision carried out by exploiting one's position or authority* that cause educational, research-related, or work-related disadvantage and worsen the environment. This includes personal attacks, refusal to provide guidance, unjustified academic disadvantage, and obstruction of research.
- **Surgery-related harassment**: in settings related to surgical care, such as surgery and postoperative management, words or actions that exceed what is necessary and appropriate for work and thereby harm the person's work environment. This category may overlap with other forms of harassment, such as power harassment. Examples include physical violence during surgery, verbal abuse during postoperative management, throwing instruments such as forceps, and unfair allocation of opportunities to serve as the operating surgeon.

* "Superior relationship" and "exploiting one's position or authority" do not refer solely to formal differences in job rank. They also include situations in which even a colleague or subordinate possesses knowledge or extensive experience necessary for work, such that duties cannot be carried out smoothly without that person's cooperation. They also include acts by a group of colleagues or subordinates in circumstances in which resistance or refusal is difficult.

* Depending on the circumstances, even conduct that would not amount to harassment if it occurred only once may still be perceived as harassment when repeated or carried out in front of many people.

** If you believe your experience overlaps across categories, such as "power harassment + surgery-related harassment," or if the distinction is difficult to make, please answer in both relevant sections.*

*Please answer the following questions based on your own experiences over the past 10 years.*

Basic Information

1. Sex (Male, Female. Other / Prefer not to answer)

2. Year of graduation from medical school (please enter the year in the Gregorian calendar)

3. Doctoral degree (Yes, No, Prefer not to answer)

4. Location of your current primary workplace

5. Which of the following best describes your current primary medical institution?

6. Which of the following best describes your current position at your primary medical institution?

Questions on Harassment (The same set of questions was applied to each category of harassment. As Questions 18 and 20 included response options representing behaviors specific to each type of harassment, the items differed across categories.)

1. Over the past 10 years, have you been subjected to harassment in general and gastroenterological surgery?

Yes

No (skip to Q14)

Do not know / Prefer not to answer (skip to Q14)

2. When did it occur? (Select all that apply.)

1-2 years after graduation (during initial residency)

3-5 years after graduation (during surgical specialty training)

6-10 years after graduation

11-20 years after graduation

21-30 years after graduation

31 or more years after graduation

Do not know / Prefer not to answer

3. At that time, which of the following best described your situation? (Select all that apply.)

Unmarried or without a partner, and without children

Unmarried or without a partner, and with children

Married or with a partner, and without children

Married or with a partner, and with children

Do not know / Prefer not to answer

4. At what type of medical institution were you working when it occurred? (Select all that apply.)

University hospital (national or public)

University hospital (private)

National Hospital Organization (NHO)

Prefectural hospital

Municipal hospital

Public hospital (e.g., Japanese Red Cross, Saiseikai)

Private hospital

Clinic

Other (please specify)

Prefer not to answer

5. How often did you experience harassment? (Select all that apply.)

Once only

Several times

10 times or more

Repeatedly / on an ongoing basis (e.g., every day)

Other (please specify)

Do not know / Prefer not to answer

6. What was the approximate age, relative to you, of the person who subjected you to harassment? (Select all that apply.)

Approximately 20 or more years older than you

Approximately 10-19 years older than you

Approximately 5-9 years older than you

Approximately the same age as you

Younger than you

Do not know / Prefer not to answer

7. What was the position of the person who subjected you to harassment? (Select all that apply.)

Chairperson / Hospital Director / Deputy Director

Department Chief / Deputy Chief

Senior Staff Surgeon / Staff Surgeon

Resident (including surgical specialty trainee)

Junior resident

Professor / Associate Professor

Lecturer / Assistant Professor

Research Fellow / Graduate Student

Other (please specify)

Do not know / Prefer not to answer

8. Where did the harassment occur? (Select all that apply.)

Ward

Outpatient clinic

Operating room

Conference

Department office / desk area

Other area within the institution

Laboratory / research room

Academic conference or other work-related travel destination

Social gathering, such as a welcome or farewell party

Do not know / Prefer not to answer

Other (please specify)

9. When did the harassment occur? (Select all that apply.)

During working hours

Outside working hours / on holidays

Do not know / Prefer not to answer

10. Which of the following applied to the power harassment you experienced, or to conduct you believed may have constituted harassment? (Select all that apply.)

Physical assault or injury (physical attacks)

Threats, defamation, insults, or severe verbal abuse (psychological attacks, including not only spoken words but also e-mail, etc.)

Isolation, exclusion, or ignoring (cutting off from interpersonal relationships)

Forcing clearly unnecessary tasks or impossible tasks, or obstructing work (excessive demands)

Assigning work far below one's abilities or experience, or not assigning work at all, without rational work-related grounds (insufficient demands)

Excessive intrusion into private matters (invasion of privacy)

Do not know / Prefer not to answer

Other (please specify)

11. When you experienced harassment, was there anyone or any office/department within your workplace that you could consult? (Select all that apply.)

There was someone I could consult, and I did so

There was an office or department I could consult, and I did so

There was someone or an office/department I could consult, but I did not or could not do so

There was no one and no office/department I could consult

Other (please specify)

Do not know / Prefer not to answer

12. If you answered "There was someone or an office/department I could consult, but I did not or could not do so" in Q11, why did you not consult? (Free-text response)

13. When you experienced harassment over the past 10 years, did you ever consider leaving the surgical profession?

Yes

No

Do not know / Prefer not to answer

14. In general and gastroenterological surgery, over the past 10 years, have you ever felt that you may have engaged in harassment?

Yes

No (skip to Q19)

Do not know / Prefer not to answer (skip to Q19)

15. When was that? (Select all that apply.)

1-2 years after graduation (during initial residency)

3-5 years after graduation (during surgical specialty training)

6-10 years after graduation

11-20 years after graduation

21-30 years after graduation

31 or more years after graduation

Do not know / Prefer not to answer

16. Over the past 10 years, how often do you think you may have engaged in conduct that may have constituted harassment? (Select all that apply.)

Once only

Several times

10 times or more

Repeatedly / on an ongoing basis (e.g., every day)

Do not know / Prefer not to answer

17. What was the approximate age, relative to you, of the person who may have been the target of the harassment you may have committed? (Select all that apply.)

Approximately 20 or more years younger than you

Approximately 10-19 years younger than you

Approximately 5-9 years younger than you

Approximately the same age as you

Older than you

Do not know / Prefer not to answer

18. Which of the following applied to conduct that you think may have constituted harassment? (Select all that apply.)

Physical assault or injury (physical attacks)

Threats, defamation, insults, or severe verbal abuse (psychological attacks, including not only spoken words but also e-mail, etc.)

Isolation, exclusion, or ignoring (cutting off from interpersonal relationships)

Forcing clearly unnecessary tasks or impossible tasks, or obstructing work (excessive demands)

Assigning work far below one's abilities or experience, or not assigning work at all, without rational work-related grounds (insufficient demands)

Excessive intrusion into private matters (invasion of privacy)

Do not know / Prefer not to answer

Other (please specify)

19. In general and gastroenterological surgery, over the past 10 years, have you ever seen or heard about harassment (excluding your own experience)?

Yes

No (skip to Q21)

Do not know / Prefer not to answer (skip to Q21)

20. Which of the following applied to harassment that you saw or heard about over the past 10 years, or to conduct you believed may have constituted harassment? (Select all that apply.)

Physical assault or injury (physical attacks)

Threats, defamation, insults, or severe verbal abuse (psychological attacks, including not only spoken words but also e-mail, etc.)

Isolation, exclusion, or ignoring (cutting off from interpersonal relationships)

Forcing clearly unnecessary tasks or impossible tasks, or obstructing work (excessive demands)

Assigning work far below one's abilities or experience, or not assigning work at all, without rational work-related grounds (insufficient demands)

Excessive intrusion into private matters (invasion of privacy)

Do not know / Prefer not to answer

Other (please specify)

21. Over the past 10 years, do you think harassment has been prevalent in general and gastroenterological surgery?

I think so

I do not think so

Do not know / Prefer not to answer

22. Do you think the Japanese Society of Gastroenterological Surgery should take measures to address harassment?

Measures should definitely be taken

Measures should be taken

I do not think measures are necessary

Do not know / Prefer not to answer

23. If you are comfortable doing so, please describe the harassment you experienced. (Free-text response)
