# Supplementary material for: Workplace Harassment and Consideration of Leaving Surgical Practice: A Nationwide Survey by the Japanese Society of Gastroenterological Surgery
Source: Ann Gastroenterol Surg. 2026 Sep 7:10.1002/ags3.70274. Online ahead of print. doi: 10.1002/ags3.70274 (PMC13550732; doi:10.1002/ags3.70274)
Supplement: Supplementary file 2 [file AGS3-9999-0-s002.docx]

Table S1: Characteristics of respondents and overall JSGS membership

|  | Respondents  n = 1967  (%) | Overall JSGS members  n = 16119  (%) |
| --- | --- | --- |
| **Sex**, male / female / prefer not to say (%) | 1711 (87.0) / 238 (12.1) / 18 (0.9) | 14546 (90.2) / 1565 (9.7) / 8 (0.1) |
| **Years since medical school graduation**, years, median [IQR^†^] | 21.0 [14.0, 29.0] | 21.0 [12.0, 31.0] |
| **Stratified by years since graduation** |  |  |
| ≤5 years  6–10 years  11–20 years  21–30 years  ≥31 years  Unknown | 51 (2.6)  265 (13.5)  666 (33.9)  587 (29.8)  398 (20.2)  0 (0) | 1351 (8.4)  2129 (13.2)  4393 (27.3)  4149 (25.7)  4088 (25.4)  9 (0.1) |

Characteristics of JSGS members aged ≤65 years are as of November 14, 2024.

†IQR: Interquartile Range
